# Effect of the COVID-19 pandemic on infants’ development: analyzing the results of developmental assessments at ages 10–11 and 18–24 months

**DOI:** 10.3389/fpsyg.2024.1430135

**Published:** 2024-09-25

**Authors:** Takashi Otani, Masaharu Kato, Hisami Haraguchi, Hideyo Goma

**Affiliations:** ^1^Department of Psychology, Faculty of Health Science, Kyoto Koka Women’s University, Kyoto, Japan; ^2^Center for Baby Science, Doshisha University, Kyoto, Japan; ^3^Department of Childcare, Kindai University Kyushu Junior College, Fukuoka, Japan; ^4^Department of Nursing, Faculty of Nursing, Himeji University, Hyogo, Japan

**Keywords:** COVID-19 pandemic, child development, individualized developmental scale, developmental questionnaire, language development

## Abstract

The purpose of this study was to determine the effect of the COVID-19 pandemic on infant development. The study investigated the development of infants at 10–11 months of age between 2020 and 2023 by using the Kyoto Scale of Psychological Development-2020 (KSPD2020), an individualized developmental scale, and the Kinder Infant Developmental Scale (KIDS), a developmental questionnaire. We compared the results of the KSPD2020 with those of a pre-pandemic developmental research and compared the developmental age (DA) of KIDS with children’s chronological age (CA). Moreover, the same developmental research was conducted again on the same children at 18–24 months of age. DA for receptive language and expressive language was lower in the KIDS compared to CA in the investigation at 10–11 months. However, in the investigation at 18–24 months, there were no areas where KIDS’ DA was lower than CA, and DA in the areas of manipulation, receptive language, social relationship with adults, discipline, and eating was higher than *CA.* On the other hand, using the KSPD2020, there were no differences when compared to pre-pandemic data in the investigation at 10–11 months. Furthermore, the investigation at 18–24 months showed that developmental quotient (DQ) was lower in the Language-Social (L-S) areas than in the investigation at 10–11 months. The lower DQ of L-S in this study was also evident in comparison to the 18–24 months pre-pandemic data. These results suggested that to investigate the medium- and long-term effects of the COVID-19 pandemic on children’s development, it is necessary to use not only parent-filled questionnaires but also individualized developmental scales. In addition, the finding that results may differ depending on the method of developmental assessment is considered important not only for developmental researchers but also for professionals involved in supporting children’s development.

## 1 Introduction

The COVID-19 pandemic has had a major impact on people’s lives. Increased anxiety concerning infection and social restrictions caused mental health problems and increased depression (Hong et al., 2021; Vindegaard and Benros, 2020). Not only adults but children and adolescents also experienced mental health problems during the pandemic (Meherali et al., 2021).

Numerous studies have indicated that the COVID-19 pandemic affected children’s development and learning. Several studies showed positive impacts of the COVID-19 pandemic. Kartushina et al. (2022) reported vocabulary growth in pandemic-exposed infants, and Finegold et al. (2023) reported that pandemic-exposed children had higher problem-solving and fine motor skills at 24 months of age (although lower in personal-social skills), as well as higher vocabulary, visual memory, cognitive performance at 54 months compared with non-exposed children. Most studies reported negative impacts. For example, Betthauser et al. (2023) reviewed 42 studies from 15 countries on the impact of school closure related with the COVID-19 pandemic on children’s learning and reported that negative effects occur early in the pandemic and persists over time. Moreover, Hagihara et al. (2022) and Hammerstein et al. (2021) also reported negative effects of school closures, indicating that these impacts occur across countries. Furthermore, reports on the effect of increased caregiver stress and anxiety concerning infection on child development (Araújo et al., 2021), the effect of wearing masks on children’s language learning and emotional understanding (Green et al., 2021; Frota et al., 2022; Giordano et al., 2024), and the negative effects of the COVID-19 pandemic are wide-ranging.

There has been significant interest in the impact of the COVID-19 pandemic on children’s language and general development, leading to numerous studies conducted in many countries. Hessami et al. (2022) conducted a meta-analysis of developmental studies using the Ages & Stages Questionnaire, Third edition (ASQ-3) in the United States, China, and Canada, comparing pandemic and pre-pandemic cohorts. The results report a higher risk of communication impairment on the pandemic cohort, compared to the pre-pandemic cohort. On the other hand, there are differences among studies in the details of the results. For example, in the United States, Shuffery et al. (2022) assessed the development of six-month-old infants using the ASQ-3, based on parent reports. They found that the scores for the gross motor, fine motor, social communication, and interpersonal communication domains reduced compared to the scores before the COVID-19 pandemic. In addition, a study conducted in Italy using the Griffiths Scales of Child Development (GSCD) reported that at 6 months of age, the pandemic data showed declines in language, communication, personal and social–emotional subscales, and overall scores (Ferrari et al., 2022). On the other hand, in China, Huang et al. (2021) conducted developmental assessments of children aged 6 months and 1 year using the ASQ-3 and Gesell Developmental Schedules (GDSs). They compared the results of the assessments from the 2015–2019 period (pre-pandemic period) with those from the January–March 2020 period (pandemic period). The rate of developmental delay at 6 months of age and 1 year of age did not differ between the pandemic and pre-pandemic periods when it was measured using the ASQ-3. However, when the GDSs were used, the rate of developmental delay increased in the fine motor and communication domains at the age of 1 year. Furthermore, Pejovic et al. (2024) revealed that long-term follow-up studies are needed because the effects on language development may not only occur immediately but also continuously. Communicative Development Inventories (CDI) and GSCD data from pre-pandemic and pandemic periods showed negative effects on language development at least up to 30 months of age. In Japan, Sato et al. (2023) reported that the effects of the COVID-19 pandemic were seen in various developmental aspects at age 5 years, but not in those younger than 3 years. Sato et al. (2023) conducted developmental assessments using the Kinder Infant Developmental Scale (KIDS) with children aged one (followed-up at age three) and those aged three (followed-up at age five) between 2017 and 2021. They compared the assessment results of a cohort that was not exposed to the COVID-19 pandemic between the two surveys with two cohorts that were exposed. Five-year-olds who were exposed to the pandemic showed developmental delays compared to those who were not exposed. In contrast, three-year-olds did not show developmental delays, regardless of whether or not they were exposed to the pandemic.

There can be at least three reasons for the discrepancy in these results. One reason could be differences in the intensity of COVID-19-related behavioral restrictions among countries and regions. During the pandemic, the United States focused on treating patients with severe illnesses or underlying diseases, and behavioral restrictions were loose. China, on the other hand, focused on the containment of COVID-19. It put fewer behavioral restrictions in areas and periods of successful containment and very strict behavioral restrictions where the infection occurred (Chen et al., 2021). In contrast, Japan implemented strict behavioral regulations almost uniformly. Another reason could be that the effect of behavioral restrictions on child development varies based on the age of the child. Behavioral restrictions that deprive children of opportunities for contact with others, such as school or nursery school closures, may affect their development. However, the degree of this effect may vary between 0 and 1-year olds, who spend a relatively high proportion of their time at home, and older children, who participate in group activities more frequently. Finally, differences in the method of conducting developmental assessments (i.e., whether a questionnaire or individualized developmental scale was used) could also explain the inconsistencies. It is very interesting that Huang et al. (2021) surveyed the same participants using questionnaire and face-to-face methods and obtained different results.

Based on the results of these studies, the current study was conducted to meet the following requirements. First, we decided to target the 0–1 year age group, which was not included in Sato et al. (2023) (who investigated the pandemic’s effect in Japan). Second, as in Huang et al. (2021), we used not only developmental questionnaire assessments but also individualized developmental scales. Finally, the study was designed to follow up on the development of the same children, considering the suggestion made by Pejovic et al. (2024) that medium- and long-term effects need to be considered. Therefore, we conducted developmental assessments of 10–11-month-old children from 2020 to 2023 and when the same children turned 18–24 months old from 2021 to 2024 using the Kyoto Scale of Psychological Development-2020 (KSPD2020), an individualized developmental scale, and the KIDS. KSPD-2020 was selected because it is widely used in Japan to assess infant development and can be administered in a short time (30–40 min) while KIDS was selected because it has been used in many studies and is suitable for comparison. With these objectives, we aimed to discuss the effect of the COVID-19 pandemic on children’s development in Japan and compare the results of individualized developmental assessments conducted by experts and developmental questionnaires completed by parents.

## 2 Methods

### 2.1 Study design

**Figure 1:**
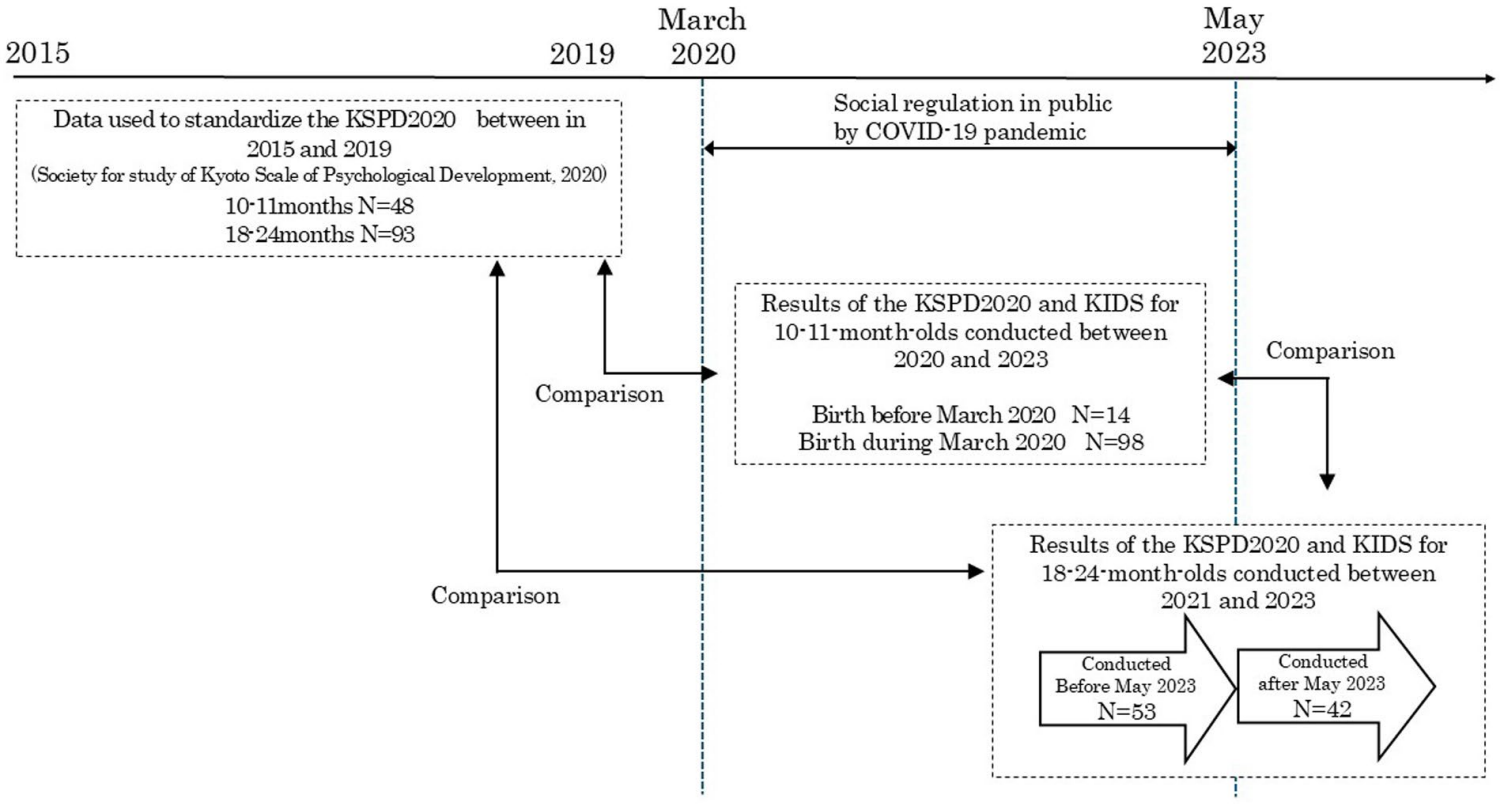
Study design.

Figure 1 illustrates the study design. We first conducted developmental assessments of children at 10–11 months of age from September 2020 to March 2023 using the KSPD2020. The results of these assessments were compared with the 2015–2019 data that was used to standardize the KSPD2020 (Society for study of Kyoto Scale of Psychological Development, 2020) to determine the effect of the COVID-19 pandemic on the development of 10–11-month-old infants (referred to as “investigation at 10–11 months”). The KIDS was administered simultaneously. The same assessments were performed when the same children were 18 months old or older (18–24-month-old children) to determine the course of their development from the age of 10–11 months to the age of 18–24 months (referred to as “investigation at 18–24 months”). These investigations were performed to identify developmental changes in infants from 10–11 to 18–24 months of age during the COVID-19 pandemic. We believed that these two investigations could shed light on the pandemic’s medium-to-long-term effect on infant development.

### 2.2 Participants

Table 1 presents the characteristics of the participants. The study included children who were born during the COVID-19 pandemic or spent at least 6 months of their early development during the pandemic. The investigation at 10–11 months comprised 112 infants (59 girls, 53 boys) assessed between October 2019 and April 2022 whose full research data were available. Six infants were born between October and December 2019, eight were born between January and February 2020, and 98 were born in March 2020 or later. Infants born before March 2020 were also included in this study because they had been under the influence of social regulation by COVID-19 pandemic for at least 6 months before the first investigation. This investigation took place from September 2020 to March 2023 and was suspended from January to March 2021, from May to June 2021, from August to September 2021, and from January to March 2022 when the government requested people to refrain from going out and engaging in social activities to prevent the spread of the infection. The participants in this investigation were aged 10–11 months (305–335 days), while their mean age was 318.2 days (SD = 9.7). They were born in the 37th week or later with a weight of at least 2,500 g and had no apparent medical conditions. The mean gestational age was 38.9 weeks (SD = 1.4), and the mean birth weight was 3110.9 g (SD = 402.9). The mean birth weight was slightly higher than the average birth weight in Japan (Ministry of Health, Labour and Welfare of Japan, 2021). This may be due to the fact that we included only full-term infants and excluded preterm infants. The mean birth weight in this study is almost consistent with the mean birth weight in studies that included only full-term infants (3,093 g) (Morisaki et al., 2016, 2017). The mean age of mothers and fathers was 33.0 years (SD = 4.0) and 35.0 years (SD = 5.7), respectively, at the time of childbirth.

**Table 1:** Characteristics of the participants.

	Investigation at 10–11 months	Investigation at 18–24 months
*N* (girls:boys)	112 (59:53)	96 (52:44)
Mean (SD) age (in days)	318.2 (9.7)	588.3 (39.2)
Age range (in days)	304–335	549–728
Mean (SD) birth weight (in grams)	3110.9 (402.9)	3121.0 (385.8)
Mean (SD) gestational weeks	38.9 (1.4)	38.9 (1.4)
Mean (SD) age of mothers (in years)	33.0 (4.0)	33.1 (4.0)
Mean (SD) age of fathers (in years)	35.0 (5.7)	34.5 (5.4)

Out of the 112 participants in the investigation at 10–11 months, the investigation at 18–24 months comprised 95 infants (52 girls, 43 boys) aged 18–24 months whose full research data were available. Fourteen infants could not participate because they had relocated or could not be contacted or declined to participate. Two infants were investigated at 18–24 months, but their full data could not be obtained. The investigation at 18–24 months was conducted from July 2021 to October 2023. It was suspended from August to September 2021 and from January to March 2022 when the government requested people to refrain from going out and engaging in social activities to prevent the spread of infection. Of the 95 investigations, 53 were conducted before March 2023, and 42 were conducted after March 2023. In Japan, social restrictions were gradually loosened before May 2023, with voluntary restrictions remaining after May 2023, from which social restrictions have been largely lifted over time. Therefore, the May 2023 time point did not represent a dramatic change in deregulation (^15^[Bibr ref002]). Consequently, in the investigation at 18–24 months, we did not separate the data by this one point but treated them as a combined total. In the investigation at 18–24 months, the mean age of the infants was 588.3 days (SD = 39.2). The average time between the investigation at 10–11 months and the investigation at 18–24 months was 270.1 days, ranging from 224 to 399 days. The mean gestational age was 38.9 weeks (SD = 1.4), and the mean birth weight was 3121.0 g (SD = 385.8). The mean age of mothers and fathers was 33.1 years (SD = 4.0) and 34.5 years (SD = 5.4), respectively, at the time of childbirth. Overall, the characteristics of the participants were similar in the two investigations.

### 2.3 Measures

#### 2.3.1 Kyoto Scale of Psychological Development-2020

The KSPD2020 is a standardized developmental scale published in 2020 that is widely used in Japan to assess child development. The KSPD-2020 has 339 test items, some of which have several sub-items. It is an individualized developmental scale that can be used among 0-year-old infants up to adults. In Japan, it is one of the few individualized developmental scales that can be administered to infants as young as 0 or 1 year old. The duration of the test is 30–40 min for infants and toddlers, and more than 1 hour for school-aged children and adults. It was standardized by collecting data from 3,243 individuals between 2015 and 2019. The validity and reliability of the scale has been confirmed using retest methods and comparison with the Wechsler Adult Intelligence Scale-Third Edition. The correlation on the retest methods was *r* = 0.69, the correlation with Wechsler Adult Intelligence Scale-Third Edition was *r* = 0.75 (Society for study of Kyoto Scale of Psychological Development, 2020). Using this tool, we calculated the overall developmental quotient (DQ) and the DQ of three development areas: Postural–Motor (P-M), Cognitive–Adaptive (C-A), and Language–Social (L-S) areas. The DQ of KSPD-2020 is calculated as the ratio of the developmental age (DA) and the chronological age (CA).

#### 2.3.2 Kinder Infant Developmental Scale

The KIDS is a standardized developmental questionnaire published in 1991 that is used to calculate a child’s developmental age for different subscales, such as physical motor and manipulation. It was standardized using data from 6,090 children between 1989 and 1990. The validity of the scale has been confirmed by comparing it with the Stanford-Binet Intelligence Scale and the Wechsler Preschool and Primary Scale of Intelligence. The correlation with the Stanford-Binet Intelligence Scale was *r* = 0.86, and the Wechsler Preschool and Primary Scale of Intelligence was *r* = 0.67 (Miyake et al., 1989; Hashimoto et al., 2013). There are three types of questionnaires according to the age of the child: Type A covers children aged 0–12 months, Type B covers children aged 1–3 years, and Type C covers children aged 3–6 years. We used Type A for the investigation at 10–11 months and Type B for the investigation at 18–24 months. Type A has six assessment areas: Physical Motor, Manipulation, Receptive Language, Expressive Language, Social relationship with Adults, and Eating. Type B has nine assessment areas, including Expressive Language, Social relationship with children, and Discipline. The KIDS can be filled by the child’s parents, the child’s nursery caregiver, or an expert (based on the answers of the parents). Each area has 13–26 question items, and respondents answer each question with a Yes or No. For example, the items of physical motor in Type A include “the child can sit up unaided,” “the child can stand for a few seconds unaided” and so on. Time needed to respond to all questions is about 10–15 min. The KSPD-2020 and KIDS differ in its structure and number of developmental areas, and do not assess the exact same aspects of development. Since both are individualized developmental scales and developmental questionnaires widely used in Japan, we believed it would be possible to evaluate the effects of the COVID-19 pandemic on children’s development from multiple perspectives by using these two tools.

### 2.4 Investigation procedure

The assessments were conducted in the research room of the Center for Baby Science, Doshisha University. This research room was equipped with a table for conducting desk-based tasks, a floor surface for turning over and crawling, and a cardboard staircase for observing infants crawling up the stairs. To prevent COVID-19 infection, this study was conducted in a constantly ventilated environment, with the research staff wearing masks with the windows of the room kept open. Despite keeping the windows open, there was no loud noise in the room as the research room did not face the roadway. The parents completed the KIDS, while experts administered the KSPD2020 to the children. The KSPD2020 was administered by professional staff members who had received adequate training in administering the scale. During the assessment, the children and their parents were present in the same room. The parents were requested not to explain the test instructions to the child or assist the child in responding. The assessments were conducted at a pre-scheduled time and lasted approximately 40–50 min, including administrative procedures for research explanation, consent, and payment of honoraria.

### 2.5 Data analysis

We conducted *t*-tests and analysis of variance (ANOVA) to determine whether there were differences in the mean DQ or DA, using IBM SPSS 26.0. For the mixed-effects models, we used R version 4.4.1 with the nlme package version 3.1–165.

### 2.6 Ethical considerations

This study was conducted with the approval of the Doshisha University Research Ethics Review Committee for Human Subjects (Approval number: 2–7), Kyoto Koka Women’s University Research Ethics Review Committee for Human Subjects (Approval number: 113), and Nara University of Education Research Ethics Review Committee for Human Subjects (Approval number: 18003). We explained the content and purpose of the study, the freedom to discontinue or decline participation, and the purpose of using and anonymizing the data to the participants verbally and in writing. Then, we obtained their voluntary consent for participation. The collected data were anonymized in a linkable manner. The participants’ names were used only when necessary, such as for the payment of rewards or deletion of data if one declined to participate.

## 3 Results

### 3.1 Investigation at 10–11 months

Regarding the results of the KSPD2020, the mean DQ was 101.3 (SD = 14.1) for P-M, 96.9 (SD = 6.8) for C-A, 96.4 (SD = 9.2) for L-S, and 97.8 (SD = 6.9) for the full scale. A one-way ANOVA of the mean DQ of the three development areas showed a significant main effect of the development area (*F* (2,222) = 10.534, *p* < 0.01, *η*^2^ = 0.087). Multiple comparisons using the Bonferroni method showed that the DQ of P-M was higher than that of C-A (*p* < 0.01, *d* = 0.402) and L-S (*p* < 0.01, *d* = 0.412). We excluded the DQ of the full scale in between-group comparisons because it included the results of the three development areas in different proportions and, unlike their DQ, was not independent.

Table 2 shows the results of the KSPD2020 for the 2015–2019 and 2020–2023 periods in the investigation at 10–11 months (Society for study of Kyoto Scale of Psychological Development, 2020). A *t*-test using the Bonferroni method was used to confirm whether the mean DQs of P-M, C-A, L-S, and the full scale in the pre-pandemic period differed significantly from those during the pandemic. No significant differences were found in the DQs of the two periods.

**Table 2:** Results of the KSPD2020 for the 2015–2019 and 2020–2023 periods at 10–11 months of age.

Results of the KSPD2020	2015–2019 (standardization data)	2020–2023 (data of this study)	*t*-test, *p*-value
*N* (girls:boys)	50 (29:21)	112 (59:53)	
Mean (SD) age (in days)	319.6 (10.5)	318.2 (9.7)	
Mean (SD) DQ of the Postural–Motor area	101.2 (13.9)	101.3 (14.1)	*t* = −0.04, *p* = 0.96
Mean (SD) DQ of the Cognitive–Adaptive area	98.7 (10.2)	96.9 (6.8)	*t* = 1.31, *p* = 0.19
Mean (SD) DQ of the Language–Social area	99.4 (13.6)	96.4 (9.2)	*t* = 1.63, *p* = 0.11
Mean (SD) DQ of the full Scale	99.4 (9.8)	97.8 (6.9)	*t* = 1.18, *p* = 0.24

**Figure 2:**
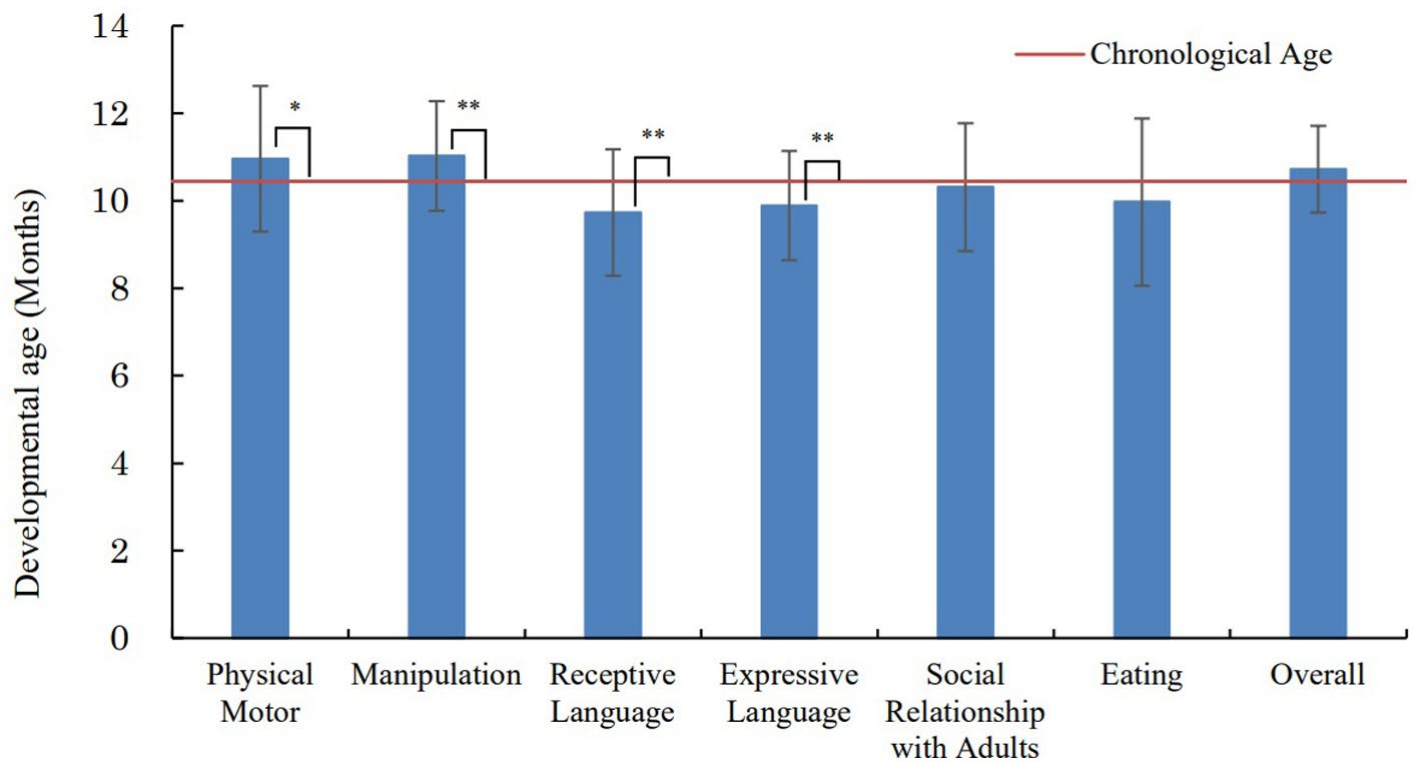
Results of the KIDS in the investigation at 10–11 months. *p < 0.05, **p < 0.01 (compared with the chronological age).

Figure 2 presents the results of the KIDS Type A in the investigation at 10–11 months. The mean DA was 11.0 months (SD = 1.7) for physical motor, 11.0 months (SD = 1.3) for manipulation, 9.7 months (SD = 1.4) for receptive language, 9.9 months (SD = 1.2) for expressive language, 10.3 months (SD = 1.5) for social relationship with adults, 10.0 months (SD = 1.9) for eating, and 10.7 months (SD = 1.0) for the overall scale. As for the KIDS there are no comparable pre-pandemic data available, a t-test using the Bonferroni method was used to confirm whether the mean DA of each assessment area and the CA at 10–11 months investigation differed significantly. The results showed that the mean DA for physical motor (*p* < 0.05, *d* = 0.426) and manipulation (*p* < 0.01, *d* = 0.626) was significantly higher than the *CA.* On the other hand, the mean DA of receptive language (*p* < 0.01, *d* = 0.691) and expressive language (*p* < 0.01, *d* = 0.617) was significantly lower than the *CA.*

### 3.2 Investigation at 18–24 months and comparing its results with those of the investigation at 10-11 months

**Figure 3:**
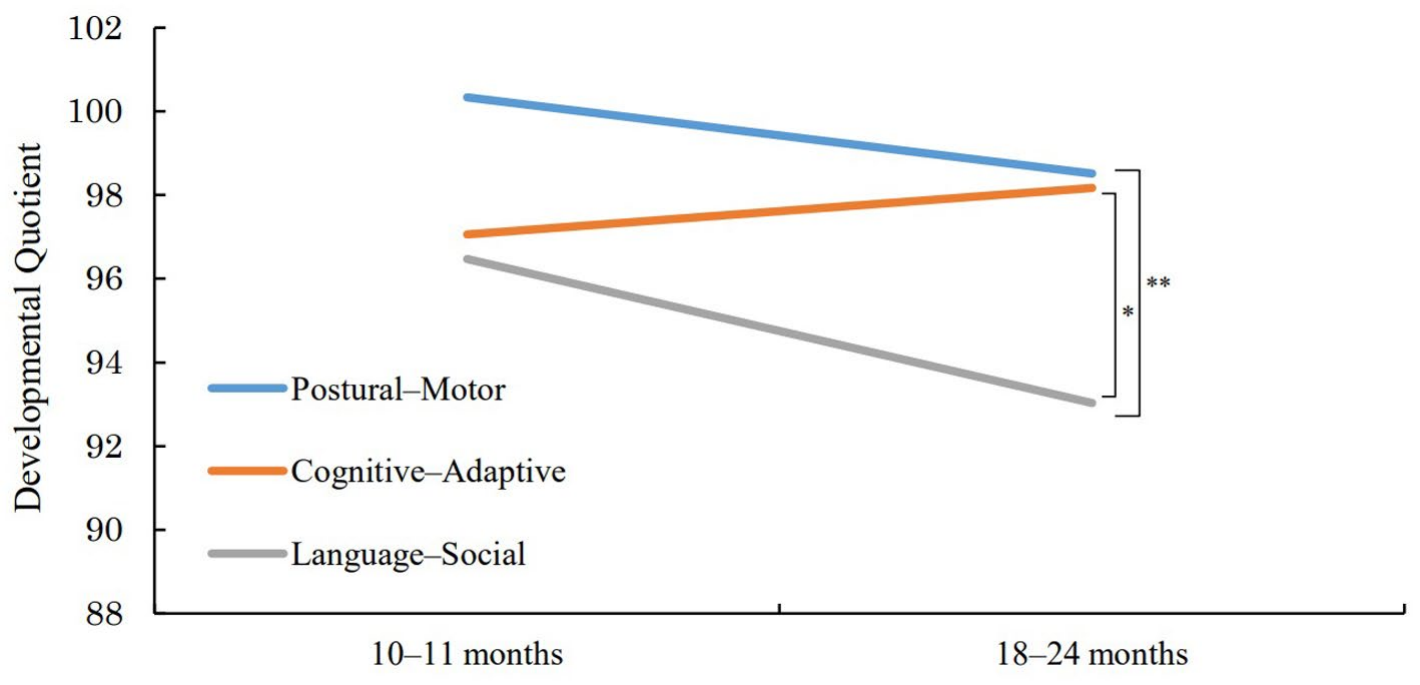
Results of KSPD at 10–11 months and 18–24 months. *p < 0.05, **p < 0.01.

Initially, the second investigation was to be conducted at 18–21 months of age. However, the period was extended to 18–24 months of age to allow more time to accommodate the research, as government restrictions due to the spread of the COVID-19 infection sometimes caused the face-to-face research to be suspended for several months. Figure 3 shows the KSPD2020 results of the 95 infants who participated in the investigation at 18–24 months. It also shows their KSPD2020 results in the investigation at 10–11 months. In the investigation at 18–24 months, the mean DQ was 98.5 (SD = 16.9) for P-M, 98.2 (SD = 13.8) for C-A, 93.0 (SD = 14.1) for L-S, and 96.7 (SD = 11.2) for the full scale. In the investigation at 10–11 months, the mean DQ was 100.3 (SD = 13.1) for P-M, 97.0 (SD = 6.9) for C-A, 96.3 (SD = 9.4) for L-S, and 97.7 (SD = 6.9) for the full scale. We constructed four mixed-effects models with increasing complexity to investigate the effects of age at the time of investigation and development areas on the developmental quotient (DQ). In all models, age at the time of investigation and development areas, along with their interaction, were treated as fixed effects, while individual differences were treated as random effects. Based on the structure of random effects, the following four models were set: Individual differences affect only the intercept (Model 1), Individual differences affect the intercept and age at the time of investigation (Model 2), Individual differences affect the intercept and development areas (Model 3), Individual differences affect the intercept, age at the time of investigation, and development areas (model 4). Model comparison using AIC (Akaike Information Criterion) showed that Model 4 had the highest goodness of fit (the AIC of lm1 was 4477.7, lm2 was 4454.3, lm3 was 4460.0, lm4 was 4420.1). Subsequently, type III Analysis of Variance (ANOVA) using Satterthwaite’s method were performed on this optimal Model 4 to assess the significance of each fixed-effects term within the model. The results of the ANOVA showed that the main effect of development areas was significant (*F* (2, 93.98) = 6.294, *p* < 0.01, *η*_p_^2^ = 0.12). Multiple comparisons using the Bonferroni method showed that the mean DQ of L-S was lower than that of P-M (*p* < 0.01, *d* = 0.424), and C-A (*p* < 0.05, *d* = 0.316). We also found an interaction effect of the age at the time of investigation and the development area (*F* (2, 187.93) = 3.177, *p* < 0.05, *η*_p_^2^ = 0.03). A simple main effect test for the interaction using the Bonferroni method showed that the mean DQ of L-S in the investigation at 18–24 months was significantly lower than that in the investigation at 10–11 months (*p* < 0.05, = 0.213). Table 3 shows the results of the KSPD2020 for the 2015–2019 and 2021–2024 periods in the investigation at 18–24 months (Society for study of Kyoto Scale of Psychological Development, 2020). A *t*-test using the Bonferroni method was used to confirm whether the mean DQs of P-M, C-A, L-S, and the full scale in the pre-pandemic period differed significantly from those during the pandemic. The results indicate that the DQ of L-S in this study was significantly lower than that from the data of 2015–2019 (*t* = 2.63, *p* < 0.05, *d* = 0.384) and that the DQ of the full scale in this study was significantly lower than that found in the data of 2015–2019 (*t* = 2.85, *p* < 0.05, *d* = 0.424).

**Table 3:** Results of the KSPD2020 for the 2015–2019 and 2021–2024 periods at 18–24 months of age.

Results of the KSPD2020	2015–2019 (standardization data)	2021–2024 (data of this study)	*t*-test, *p*-value
*N* (girls:boys)	93 (45:48)	95 (53:42)	
Mean (SD) age (in days)	641.7 (42.1)	588.3 (39.22)	
Mean (SD) DQ of the Postural–Motor area	102.1 (9.7)	98.5 (17.0)	*t* = 1.39, *p* = 0.17
Mean (SD) DQ of the Cognitive–Adaptive area	102.1 (18.4)	98.2 (13.9)	*t* = 2.26, *p* = 0.08
Mean (SD) DQ of the Language–Social area	98.1 (12.4)	93.0 (14.1)	*t* = 2.63, *p* < 0.05
Mean (SD) DQ of the full Scale	100.8 (8.0)	96.7 (11.3)	*t* = 2.85, *p* < 0.05

**Figure 4:**
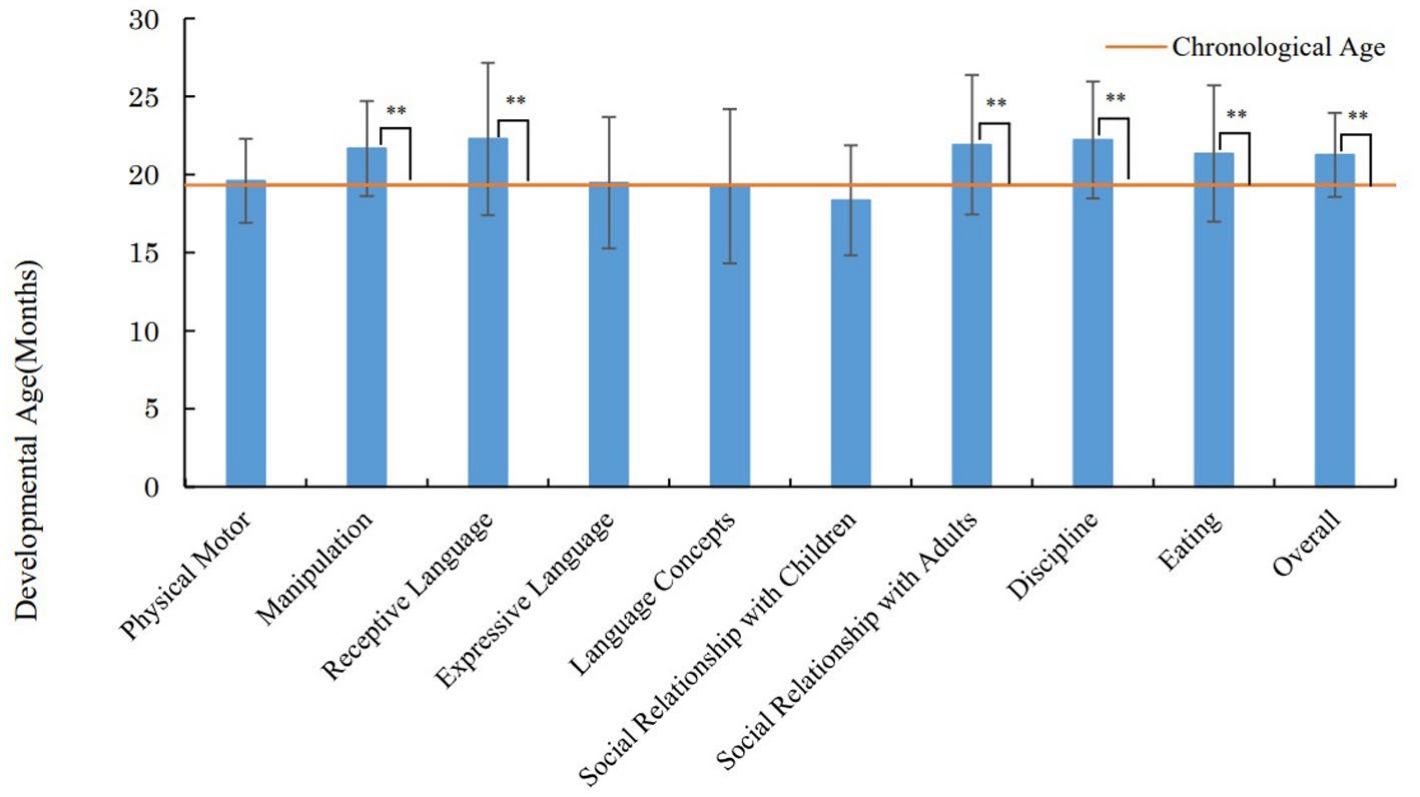
Results of the KIDS in the investigation at 18–24 months. **p < 0.01 (compared with the chronological age).

Figure 4 presents the results of the KIDS Type B in the investigation at 18–24 months. The mean DA was 19.6 months (SD = 2.7) for physical motor, 21.6 months (SD = 3.0) for manipulation, 22.3 months (SD = 4.9) for receptive language, 19.5 months (SD = 4.2) for expressive language, 19.3 months (SD = 4.9) for language concepts, 18.4 months (SD = 3.5) for social relationship with children, 21.8 months (SD = 4.5) for social relationship with adults, 22.2 months (SD = 3.8) for discipline, 21.0 months (SD = 5.3) for eating, and 21.3 months (SD = 2.7) for the overall scale. A *t*-test using the Bonferroni method was used to confirm whether the mean DA of each assessment area and the CA at 18–24 months investigation differed significantly. The results showed that the mean DA for manipulation (*p* < 0.01, *d* = 1.019), receptive language (*p* < 0.01, *d* = 0.853), social relationship with adults (*p* < 0.01, *d* = 0.748), discipline (*p* < 0.01, *d* = 1.039) and eating (*p* < 0.01, *d* = 0.657) was significantly higher than that of the *CA.* Table 4 shows that the difference between the DA in 6 areas (physical motor, manipulation, receptive language, expressive language, social relationship for adults, and eating) in the 10–11 months investigation and the 18–24 months investigation and the DA was calculated and compared to the difference in CA in these two investigations (Table 4). As results, the DA differences in the areas of manipulation (*p* < 0.01, *d* = 0.770), receptive language (*p* < 0.01, *d* = 1.096), social relationship for adults (*p* < 0.01, *d* = 0.838), and eating (*p* < 0.01, *d* = 0.832) were larger than that of the *CA.*

**Table 4:** Result of KIDS for the investigation at 10–11 months and the investigation at 18–24 months.

Developmental areas	Differences in CA between the investigation at 10–11 months and 18–24 months	Differences in DA between the investigation at 10–11 months and 18–24 months	*t*-test, *p*-value
Physical motor	8.91 (1.29)	8.73 (2.30)	*t* = 0.08, *p* = 0.42
Manipulation	8.91 (1.29)	10.71 (3.04)	*t* = 6.30, *p* < 0.01
Receptive language	8.91 (1.29)	12.75 (4.79)	*t* = 8.16, *p* < 0.01
Expressive language	8.91 (1.29)	9.78 (4.20)	*t* = 2.15, *p* = 0.21
Social relationship with adults	8.91 (1.29)	11.56 (4.29)	*t* = 6.52, *p* < 0.01
Eating	8.91 (1.29)	11.60 (4.39)	*t* = 6.21, *p* < 0.01

## 4 Discussion

The results of the investigation at 10–11 months using the KIDS showed that the DA of receptive language and expressive language were significantly lower than that of the *CA.* The results are similar to those of Hessami et al. (2022) and Ferrari et al. (2022) in that the developmental questionnaire survey revealed delayed language development in infants younger than 1 year of age. On the other hand, no differences were found in the investigation at 10–11 months upon using the KSPD-2020. In contrast, results of the investigation at 18–24 months using the KIDS showed no significant difference. However, the DQ of L-S using the KSPD-2020 in this study was lower than pre-pandemic data. Furthermore, the fact that different results were obtained depending on whether a questionnaire or an individual developmental scale was used aligns with findings from of Huang et al. (2021). However, while Huang et al. (2021) confirmed delays in fine motor and communication functions, this study did not find delays in the development of cognitive aspects involving fine motor function. This discrepancy may have been due to differences in COVID-19-related behavioral restrictions and child-rearing environments in Japan and China.

This study had some limitations. In this study, the examiner wore a mask while conducting the KSPD-2020, unlike in the 2015–2019 investigation. In the investigation at 10–11 months and 18–24 months in this study, the same condition of wearing masks was observed but differences in DQ of L-S occurred in the investigation at 10–11 months and 18–24 months. Although it is difficult to attribute the decline in DQ of L-S at 18–24 months solely to the wearing of masks at the time of the investigation, we cannot completely rule out the possibility that masks affected the scores. In addition, for the 18–24 months investigation, it was necessary to conduct the investigation promptly at 18 months to avoid suspension of the investigation due to the spread of the COVID-19 in this study. Therefore, the sampling was not identical to the 2015–2019 time point, and there was a gap between the average of CA in the two sets of data (2015–2019: 641.7 days, 2021–2024: 588.3 days). The large age range in the second investigation and the unequal sampling within this range were limitations of this study.

It is possible that differences not only in the assessment method but also in the method of comparison may have influenced the difference in the presence or absence of pandemic effects, depending on whether the investigation was conducted using a developmental questionnaire or an individualized developmental scale. For the KSPD-2020, it was possible to compare data from the 2015–2019 pre-pandemic, but since there were no pre-pandemic data for the KIDS, the study was based on comparisons with the *CA.*

In this study, the developmental course of the same children was followed and investigated from 10 to 11 months of age to 18–24 months of age. These data allowed us to confirm the subsequent developmental course of children who were born during or spent their early developmental years under the pandemic. The KIDS results indicate that although DA in receptive language and expressive language was lower than CA in the investigation at 10–11 months, development tended to be accelerated relative to CA in most aspects of development from 10–11 to 18–24 months of age, and by 18–24 months of age, the DA of expressive language was no longer different from the CA and the DA of receptive language exceeded *CA.* The KSPD-2020 results showed a delay from the pre-pandemic data that was not seen at 10–11 months of age, but was confirmed in the investigation at 18–24 months of age. These results suggest that the effects of the COVID-19 pandemic on development should be discussed based on multiple measures and results at multiple time points, and confirm the importance of follow-up studies on the same children, as Pejovic et al. (2024) suggested.

The difference in the results of this study depending on whether individualized developmental scales or developmental questionnaires were used is an important finding, confirming results from Huang et al. (2021). Additionally, our results complement Sato et al.’s (2023) finding that the effects on language development start from a younger age. This can serve as a valuable resource in examining the effects of the COVID-19 pandemic on the development of Japanese children. A detailed investigation should be conducted on the effects of the COVID-19 pandemic on infant development by using not only a questionnaire but also individual observations by experts. The fact that the results differed between the individualized developmental scales and the developmental questionnaires is an important finding not only for developmental researchers but also for professionals involved in developmental support. It is important to keep in mind the limitations of the scales and questionnaires, and to use them with the understanding that the aspects of a child’s development one can assess vary depending on the tests. It should be noted that communication problems may be less likely to surface with family members who are involved on a daily basis.

Furthermore, it is of great concern to us how the 1-year-old infants who participated in this study will develop when they enter kindergarten and nursery school. It is possible that immaturity in communication will persist with non-family members even after growth. However, the effects may be ameliorated by the lifting of social restrictions and by the age at which they experience group living (e.g., preschool age). It is thus necessary to investigate the medium- and long-term effects, as Pejovic et al. (2024) suggested that effects on language development may continue until later. Considering that child development should continue to be investigated, developmental assessments are being conducted on the infants who participated in this study and turned 3 years old.

## Data Availability

The datasets presented in this article are not readily available because the dataset cannot be provided to third parties because participants’ consent has not been obtained for any purpose other than the original study. However, if a situation arises in which objective confirmation of the research content is required, we are prepared to disclose the data for appropriate confirmation. Requests to access the datasets should be directed to Takashi Otani, t-otani@mail.koka.ac.jp.
